# Physicochemical Salt Solution Parameters Limit the Survival of *Planococcus halocryophilus* in Martian Cryobrines

**DOI:** 10.3389/fmicb.2020.01284

**Published:** 2020-07-07

**Authors:** Annemiek C. Waajen, Jacob Heinz, Alessandro Airo, Dirk Schulze-Makuch

**Affiliations:** ^1^UK Centre for Astrobiology, School of Physics and Astronomy, The University of Edinburgh, Edinburgh, United Kingdom; ^2^Astrobiology Research Group, Center of Astronomy and Astrophysics, Technische Universität Berlin, Berlin, Germany; ^3^School of the Environment, Washington State University, Pullman, WA, United States; ^4^Section Geomicrobiology, GFZ German Research Centre for Geosciences, Potsdam, Germany; ^5^Department of Experimental Limnology, Leibniz Institute of Freshwater Ecology and Inland Fisheries (IGB), Stechlin, Germany

**Keywords:** *Planococcus halocryophilus*, salt stress, water activity, ionic strength, survival

## Abstract

Microorganisms living in sub-zero environments can benefit from the presence of dissolved salts, as they significantly increase the temperature range of liquid water by lowering the freezing point. However, high concentrations of salts can reduce microbial growth and survival, and can evoke a physiological stress response. It remains poorly understood how the physicochemical parameters of brines (e.g. water activity, ionic strength, solubility and hydration shell strength between the ions and the surrounding water molecules) influence the survival of microorganisms. We used the cryo− and halotolerant bacterial strain *Planococcus halocryophilus* as a model organism to evaluate the degree of stress different salts assert. Cells were incubated in liquid media at −15°C containing single salts at eutectic concentrations (CaCl_2_, LiCl, LiI, MgBr_2_, MgCl_2_, NaBr, NaCl, NaClO_4_ and NaI). Four of these salts (LiCl, LiI, MgBr_2_ and NaClO_4_) were also investigated at concentrations with a low water activity (0.635) and, separately, with a high ionic strength (8 mol/L). Water activity of all solutions was measured at −15°C. This is the first time that water activity has been measured for such a large number of liquid salt solutions at constant sub-zero temperatures (−15°C). Colony-Forming Unit (CFU) counts show that the survival of *P. halocryophilus* has a negative correlation with the salt concentration, molecular weight of the anion and anion radius; and a positive correlation with the water activity and anions’ hydration shell strength. The survival of *P. halocryophilus* did not show a significant correlation with the ionic strength, the molecular weight of the cation, the hydrated and unhydrated cation and hydrated anion radius, and the cations’ hydration bond length. Thus, the water activity, salt concentration and anion parameters play the largest role in the survival of *P. halocryophilus* in concentrated brines. These findings improve our understanding of the limitations of microbial life in saline environments, which provides a basis for better evaluation of the habitability of extraterrestrial environments such as Martian cryobrines.

## Introduction

Liquid water is a requirement for life as we know it. Dissolved salts can depress the freezing point of water significantly. As this increases the temperature range of liquid water, this expands the habitable zone around stars (Kasting et al., 1993) and broadens the range of habitable environments on planets such as Mars.

Even though the long-term presence of liquid water on the surface of Mars is not possible due to the lack of a sufficiently dense atmosphere, liquid water could be temporarily stable under current Martian surface conditions as cryobrines, i.e., aqueous salty solutions with a eutectic temperature below 0°C (Möhlmann and Thomsen, 2011). The eutectic temperature is the lowest freezing temperature that can be obtained with a respective salt. This, in combination with the discovery of perchlorates on Mars (Hecht et al., 2009), suggests that perchlorate-rich brines could be present on Mars nowadays (Kereszturi et al., 2010; Chevrier and Rivera-Valentin, 2012; Martín-Torres et al., 2015; Ojha et al., 2015).

The existence of Martian cryobrine environments is supported by the recent putative discovery of a 1.5 km deep subsurface lake (Orosei et al., 2018). Although the lake water is assumed to have a temperature of −68°C, it remains presumably liquid because of its high concentration of dissolved salts (Fisher et al., 2010; Orosei et al., 2018). Furthermore, Martian Recurring Slope Lineae (RSL), i.e., seasonal dark streaks on steep slopes that slowly appear in spring and vanish in late summer (McEwen et al., 2011), have been suggested to be caused by the formation of surface cryobrines (Kereszturi et al., 2010; Chevrier and Rivera-Valentin, 2012; Ojha et al., 2015). This could indicate that potentially habitable cryobrine environments could exist temporarily near the Martian surface, supposedly containing sodium, calcium, or magnesium chlorides or perchlorates (Chevrier et al., 2009; McEwen et al., 2011).

The habitability of Martian cryobrines can be assessed through the study of extremotolerant organisms adapted to cold and saline environments on Earth, such as the halocryotolerant bacterium *Planococcus halocryophilus*. *P. halocryophilus* was isolated from Canadian permafrost soil and is capable of growth in 19 wt/vol% NaCl solution at −15°C (Mykytczuk et al., 2012, 2013). Furthermore, it has the highest bacterial perchlorate tolerance (13.6 wt/vol% NaClO_4_ at +25°C) reported to date (Heinz et al., 2019). Only some fungi are known to tolerate higher perchlorate concentrations (Heinz et al., 2020). Hence, this halo- and cryotolerant organism is highly suitable as a model organism for studying the habitability of Martian cryobrines and was therefore used in this study.

Independent of such species-specific adaptations, all microorganisms are known to respond to salt stress through various molecular biological processes ranging from active transmembrane ion transport to dormancy. If a certain salt type or concentration generates a biochemical stress response, numerous defects can occur, such as protein denaturation or membrane damage, eventually leading to cell death. Deciphering the mechanisms by which each ion impedes microbial growth or survival is challenging, due to a potential overlap of different mechanisms that can additionally be highly species-dependent. At best, the toxicity of an ion correlates with a single physicochemical parameter either relating to the ion itself (e.g., ionic radius) or affected indirectly by the ion concentration (e.g., water activity). However, additional toxicities such as chemical reactivity (e.g., the reducing effect of ions like iodide) or biochemical toxicity [e.g., the interaction of calcium on extracellular polymeric substance (EPS)] can also influence microbial survival.

Research on the physicochemical parameters limiting the habitability of brines has largely focused on water activity, which is a measure of its thermodynamic availability (Tosca et al., 2008; Stevenson et al., 2015a, b). The maximum degree to which a salt can reduce the water activity also depends on its solubility, which is related to the hardness of the involved ions, which in turn derives from their charge/radius ratio. Ionic strength however, which is a measure for the strength of the electric field in a solution, has received less attention regarding the survival of microorganisms (Fox-Powell et al., 2016).

Several physicochemical parameters are influenced by the hardness of ions. The hardness of an ion is determined by the charge density of the respective ion, which is influenced by the charge/radius ratio of that ion. Hard ions have a high charge density, while soft ions have a low charge density. The Hard and Soft Acids and Bases (HSAB) theory states that salts consisting of hard cations and soft anions or vice versa are more soluble than salts consisting of either hard cations and hard anions or soft cations and soft anions (Pearson, 1968a,b). Furthermore, a higher solubility correlates with a lower minimal water activity in a saturated solution, as more water molecules are needed to dissolve the increased number of ions in the more concentrated solution. As water activity is one of the main parameters expected to influence microbial survival, the higher solubility, and thus the HSAB theory, might describe microbial survival. The link between these physicochemical parameters in light of the HSAB theory is investigated in this paper.

**FIGURE 1:**
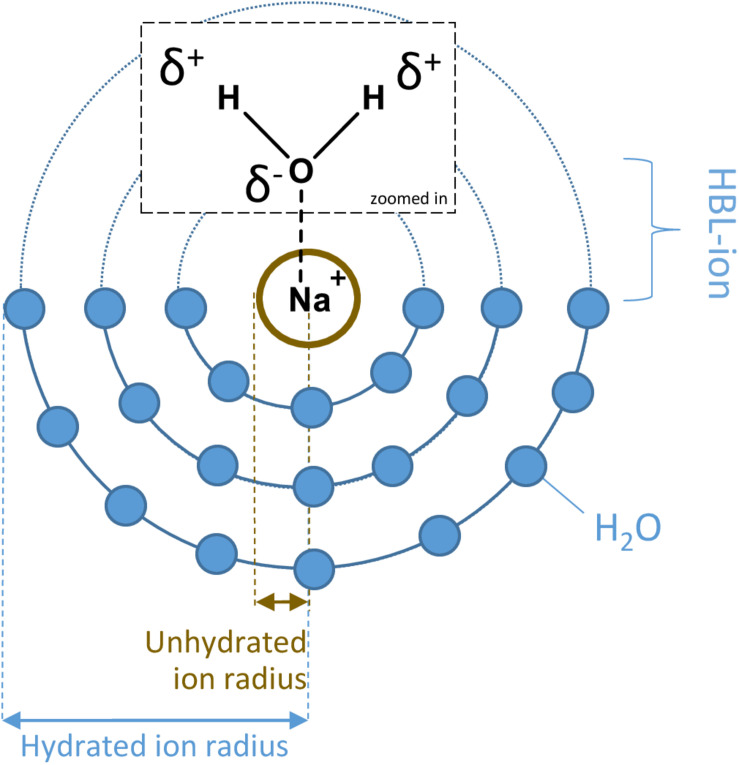
An example of hydration spheres surrounding a sodium-ion in solution, including a depiction of the unhydrated and hydrated ion radius. The HBL-ion is the hydrogen bond length between the ion and the oxygen atom of the water molecules in the inner hydration shell surrounding the ion.

Heinz et al. (2018) investigated the survival of *P. halocryophilus* in various chloride and perchlorate solutions at different temperatures. The study showed that decreasing the temperature under high salt concentrations results in a higher survival of *P. halocryophilus*. The hypothesis for the higher survival at sub-zero temperatures is that besides the normal Arrhenius-like temperature dependence, the size and stability of hydration shells around the salt ions, which increases with decreasing temperatures, reduces the osmotic stress (Heinz et al., 2018). The size and stability of hydration shells depend on the hydration bond length (HBL) between the ion and the oxygen atom of the water molecules in the inner hydration shell (HBL-ion), which is determined by the charge and the radius of the ion (Figure 1). The radii of the ions taken into account in this study are the unhydrated or crystal ion radii and the hydrated or effective ion radii (Nightingale, 1959). As the size and stability of hydration shells differ between salts, this could influence microbial survival in brines containing these salts due to, for example, differences in ion transportation across the cell membrane.

It remains unknown which physicochemical parameters of a salt solution have the greatest influence on the survival of *P. halocryophilus* and whether additional chemical reactivity or biochemical toxicity plays a role. A recent study by Heinz et al. (2019) suggests for chloride and perchlorate salt solutions that ion-specific factors of salts have a larger influence on the growth limitation of *P. halocryophilus* than general physicochemical parameters of the solution, such as water activity or ionic strength. In order to determine whether this applies to other salts, and to establish the relevance of other physicochemical salt parameters, we investigated the influence of a wide range of physicochemical parameters on the survival of *P. halocryophilus*. The investigated physicochemical parameters include water activity, ionic strength, HBL between the salt ions and water molecules, hydrated and unhydrated ion radii, salt concentration and ion molecular weight. Although an often-mentioned physicochemical salt parameter, chaotropicity of the ions could not be investigated due to the lack of quantitative data for most of the investigated salts.

## Materials and Methods

*Planococcus halocryophilus* Or1 (DSMZ 24743) was grown in liquid DSMZ #92 medium (30 g/L TSB; 3 g/L yeast) at +25°C in batch incubations without shaking. Cultures were transferred weekly to fresh medium. Death rate experiments were performed as described by Heinz et al. (2018). Briefly, 2 mL of *P. halocryophilus* Or1 cell suspension of growth medium +10 wt% NaCl at late exponential phase was stored at 4°C for 30–60 min and subsequently added to 8 mL of −15°C salt solution, resulting in a final salt concentrations as described in Tables 1, 2. The death rate of *P. halocryophilus* was determined by plating sample aliquots and Colony-Forming Unit (CFU) determination. Death rate is defined as the decrease in CFU count per minute. CFU counts were plotted over time and the data was fitted with a logarithmic regression. For CFU determinations, culture samples were taken at multiple time points and diluted with −15°C phosphate-buffered saline (PBS) + 20 wt% NaCl (7 g/L Na_2_HPO_4_.2H_2_O; 3 g/L KH_2_PO_4_; 250 g/L NaCl). Serial dilution was done in 1.5 mL reaction tubes with 900 μL PBS + 20% NaCl, followed by plating on 4°C DSMZ growth medium #92 agar plates containing no additional salt (30 g/L TSB; 3 g/L yeast; 15 g/L agar). Agar plates were incubated at +25°C. All experiments were either performed in technical and biological duplicates, or in technical triplicates.

**TABLE 1:** Chemical salt parameters at eutectic concentration for death rate experiments.

	Eutectic concentration		Salt concentration dependent parameters			Salt concentration independent parameters						Death rate results
				
Salt	Concen-tration (wt%)	Molar concen-tration (mol/L)	Measured water activity at −15°C ± standard deviation	Calculated water activity at +25°C	Ionic strength (mol/L)	Hydrated cation radius^a^ (Å)	Unhydrated cation radius^a^ (Å)	Hydrated anion radius^a^ (Å)	Unhydrated anion radius^a^ (Å)	HBL-cation^a^ (Å)	HBL-anion^a^ (Å)	Death rate (min^–1^)
CaCl_2_	30.2^c^	3.90	0.5730 ± 0.0024	0.64	11.70	4.12	0.99	3.32	1.81	2.46^h^	3.21^h^	2.0 × 10^–5^
LiCl	25.3^c^	7.99	0.4537 ± 0.0053	0.54	7.99	3.82	0.68	3.32	1.81	1.94^i^	3.21^h^	6.4 × 10^–4^
LiI	51.5^e^	7.93	0.3941 ± 0.0033	0.39	7.93	3.82	0.68	3.31	2.16	1.94^i^	3.55^i^	6.6
MgBr_2_	37.0^f^	3.19	0.5942 ± 0.0019	0.66	9.57	4.28	0.65	3.30	1.95	2.10^h^	3.47^j^	8.6 × 10^–4^
MgCl_2_	21.0^b^	2.79	0.6600 ± 0.0132	0.75	8.38	4.28	0.65	3.32	1.81	2.10^h^	3.21^h^	1.5 × 10^–5^
NaBr	40.3^g^	6.56	0.6490 ± 0.0049	0.71	6.56	3.58	0.95	3.30	1.95	2.43^i^	3.47^j^	1.9 × 10^–4^
NaCl	23.3^b^	5.20	0.7283 ± 0.0019	0.80	5.20	3.58	0.95	3.32	1.81	2.43^i^	3.21^h^	3.7 × 10^–6^
NaClO_4_	53.0^g^	9.06	0.6422 ± 0.0082	0.68	9.06	3.58	0.95	3.38	2.92	2.43^i^	3.68^k^	3.3 × 10^–2^
NaI	47.1^g^	5.94	0.6758 ± 0.0070	0.72	5.94	3.58	0.95	3.31	2.16	2.43^i^	3.55^i^	6.0 × 10^–1^

***^*φ*^**Hydration bond lengths between ions and oxygen atom of the water molecule in solution at +25°C. Water activity values were measured at −15°C and calculated for +25°C. Ionic strength values are calculated with the Pitzer equations (Pitzer, 1973, 1975, 1991). References: ^*a*^(Nightingale, 1959), ^*b*^(Heinz et al., 2018), ^*c*^(Conde, 2004), ^*d*^(Madan, 2012), ^*e*^(Hendricks, 1928), ^*f*^(Hennings, 2014), ^*g*^(Fegley and Osborne, 2013), ^*h*^(Bruni et al., 2012), ^*i*^(Mähler and Persson, 2012), ^*j*^(Kálmán et al., 1983), ^*k*^(Lindqvist-Reis et al., 1998).*

**TABLE 2:** Chemical salt parameters at non-eutectic concentrations for death rate experiments.

	Non-eutectic concentration		Salt concentration dependent parameters			Salt concentration independent parameters						Death rate results
				
Salt	Concen-tration (wt%)	Molar concen-tration (mol/L)	Measured water activity at −15°C ± standard deviation	Calculated water activity at +25°C	Ionic strength (mol/L)	Hydrated cation radius^a^ (Å)	Unhydrated cation radius^a^ (Å)	Hydrated anion radius^a^ (Å)	Unhydrated anion radius^a^ (Å)	HBL-cation^a^ (Å)	HBL-anion^a^ (Å)	Death rate (min^–1^)
LiCl	19.0	5.53	0.6157 ± 0.0033	0.70	5.53	3.82	0.68	3.32	1.81	1.94^b^	3.21^c^	2.8 × 10^–4^
LiI	39.4	4.86	0.6333 ± 0.0027	0.70	4.86	3.82	0.68	3.31	2.16	1.94^b^	3.55^b^	2.3 × 10^–1^
MgBr_2_	35.0	2.92	0.6268 + 0.0100	0.70	8.77	4.28	0.65	3.30	1.95	2.10^c^	3.47^d^	2.7 × 10^–3^
NaClO_4_	51.3	8.60	0.6539 ± 0.0029	0.70	8.60	3.58	0.95	3.38	2.92	2.43^b^	3.68^e^	6.0 × 10^–2^
LiCl	25.3	7.99	0.4537 ± 0.0053	0.54	7.99	3.82	0.68	3.32	1.81	1.94^b^	3.21^c^	7.6 × 10^–4^
LiI	51.7	8.00	0.3941 + 0.0033	0.38	8.00	3.82	0.68	3.31	2.16	1.94^b^	3.55^b^	6.6
MgBr_2_	32.9	2.66	0.6632 + 0.0032	0.74	7.99	4.28	0.65	3.30	1.95	2.10^c^	3.47^d^	3.1 × 10^–3^
NaClO_4_	49.5	8.01	0.6732 ± 0.0114	0.72	8.01	3.58	0.95	3.38	2.92	2.43^b^	3.68^e^	6.4 × 10^–2^

***^*φ*^**Hydration bond lengths between ions and oxygen atom of water in solution at +25°C. Water activity values were measured at −15°C and calculated for +25°C. Ionic strength values are calculated with the Pitzer equations (Pitzer, 1973, 1975, 1991). References: ^*a*^(Nightingale, 1959), ^*b*^(Mähler and Persson, 2012), ^*c*^(Bruni et al., 2012), ^*d*^(Kálmán et al., 1983), ^*e*^(Lindqvist-Reis et al., 1998).*

The death rate of *P. halocryophilus*, calculated from the CFU counts, was investigated at the eutectic salt concentrations of the following salts: CaCl_2_, LiCl, LiI, MgBr_2_, MgCl_2_, NaBr, NaCl, NaClO_4_ and NaI. Death rate data in NaCl, CaCl_2_ and MgCl_2_ at eutectic concentrations were obtained by Heinz et al. (2018) using the same methodology as in this study. Additionally, death rates in LiCl, LiI, MgBr_2_ and NaClO_4_ were investigated at a water activity of 0.635 ± 0.02 at −15°C and separately at an ionic strength of 8 mol/L. The salt concentrations and physicochemical parameters at the tested conditions are shown in Tables 1, 2. Water activity was measured in triplicate at −15°C using a humidity and temperature probe (HC2-AW, ROTRONIC Instruments (UK) Ltd, Crompton Fields). Three milliliter of salt solution was stored in the probe and the water activity was measured for several hours until stabilization. Additionally, the water activity of the solutions at +25°C were calculated using the Pitzer equation to compare these values with the measured water activity values at −15°C (Pitzer, 1973, 1975, 1991). Pitzer equation parameters were taken from Kim and Frederick (1988). The correlations between the death rate of *P. halocryophilus* and physicochemical parameters were determined with the help of univariate linear regression. The correlation coefficients of univariate linear regressions were all obtained from logarithmic regression lines.

## Results

### Experiments at Eutectic Concentrations

#### Salt Concentration Dependent Parameters

**FIGURE 2:**
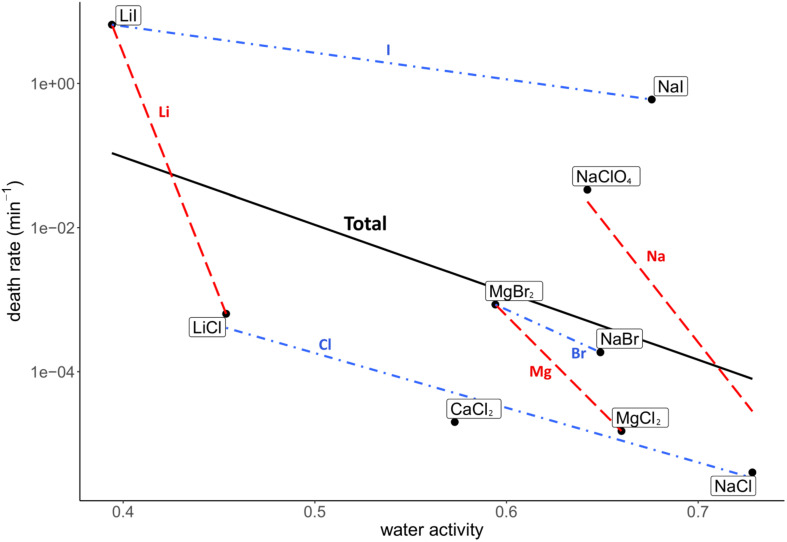
Correlation between the death rate and water activity at −15°C at eutectic concentration. Regression of all salts is depicted in black (*R*^2^ = 0.23). Regressions of salts with the same cations are depicted with red dashed lines, regressions of salts with the same anion are in blue dashdotted lines. The overall negative correlation is supported by the trends of salts with the same anion and cation, which all show a negative trend.

**FIGURE 3:**
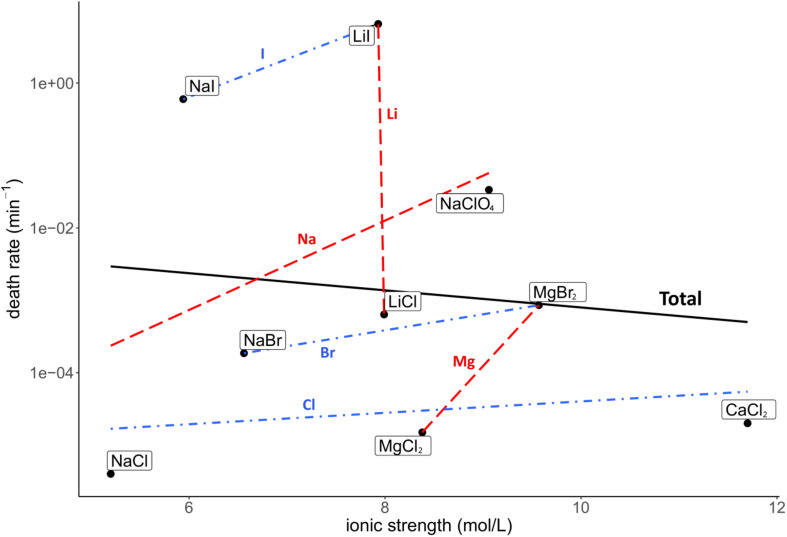
Correlation between the death rate and ionic strength at eutectic concentration. Regression of all salts is depicted in black (*R*^2^ = 0.01). Regressions of salts with the same cations are depicted with red dashed lines, regressions of salts with the same anion are in blue dashdotted lines. No overall correlation is seen, nor do the trend lines of salts with the same anion and cation show an indication of a correlation.

**FIGURE 4:**
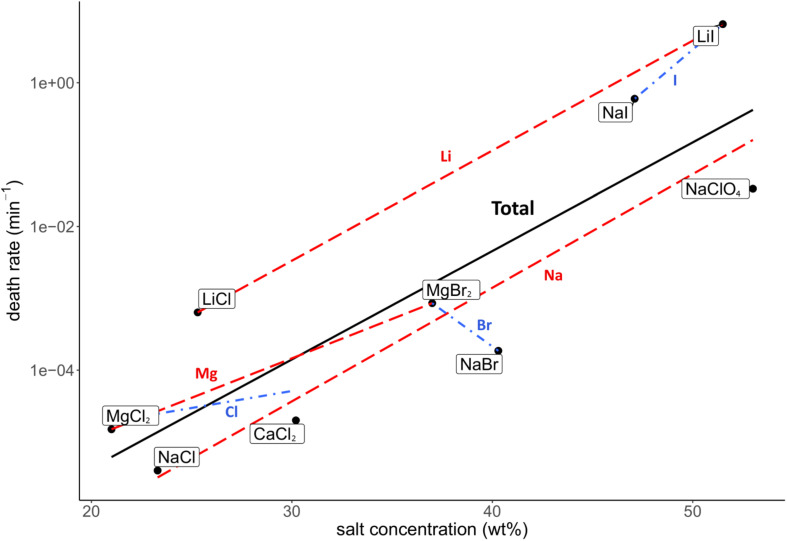
Correlation between the death rate and salt concentration (wt%) at eutectic concentration. Regression of all salts is depicted in black (*R*^2^ = 0.74). Regressions of salts with the same cations are depicted with red dashed lines, regressions of salts with the same anion are in blue dashdotted lines. The overall strong, positive correlation is supported by the trends of salts with the same cation, which all show a positive trend. However, salts with the same anion show no trends.

Death rate and physicochemical salt parameter values at eutectic concentrations at −15°C are presented in Table 1. The quality of the correlations of parameters with the death rates are presented as *R*^2^ values. The strength of the correlations is dependent on the slope of the correlation. The quality of the logarithmic regression of CFU counts determining the death rate at eutectic concentrations was high, as *R*^2^ was higher than 0.8 for all salts except for MgBr_2_, which had a lower quality (*R*^2^ = 0.46) (data not shown). The investigated salt concentration dependent parameters were water activity and ionic strength. Salt concentration itself was also investigated. A negative correlation between the death rate and the water activity at −15°C was observed (*R*^2^ = 0.23; Figure 2). This negative correlation was confirmed when comparing salts with the same cation or anion (red and blue linear regression lines in Figure 2, respectively). No correlation was observed between the death rate and the ionic strength of the solution at the eutectic concentration (*R*^2^ = 0.01; Figure 3). Moreover, there are no trends indicating a correlation for salts with the same cation or anion. A strong, positive correlation was found between salt concentrations and death rate (*R*^2^ = 0.74; Figure 4), which was confirmed with positive trends in salts with the same cation, but not with the same anion.

#### Salt Concentration Independent Parameters

**FIGURE 5:**
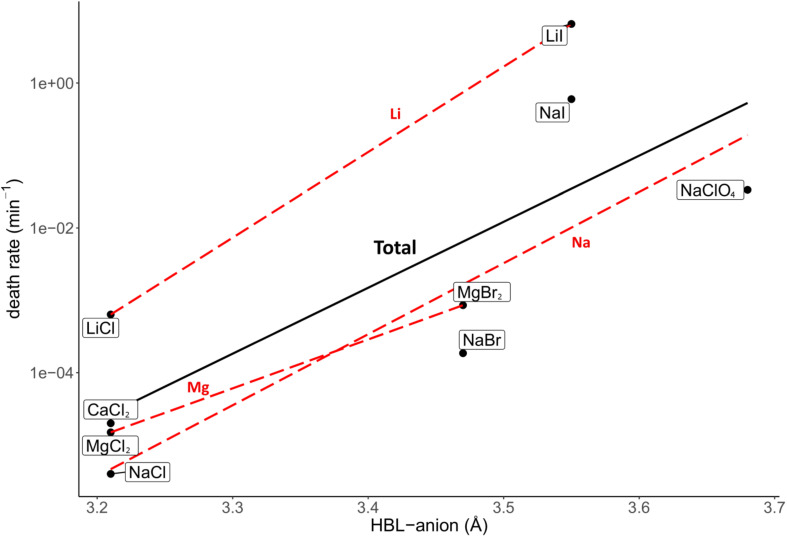
Correlation between the death rate at eutectic concentration and hydration bond length between the anion and the oxygen atoms of the water molecules in the inner hydration shell (HBL-anion). Regression of all salts is depicted in black (*R*^2^ = 0.62). Regressions of salts with the same cations are depicted with red dashed lines. The overall strong, positive correlation is supported by the trends of salts with the same anion, which all show a positive trend.

**FIGURE 6:**
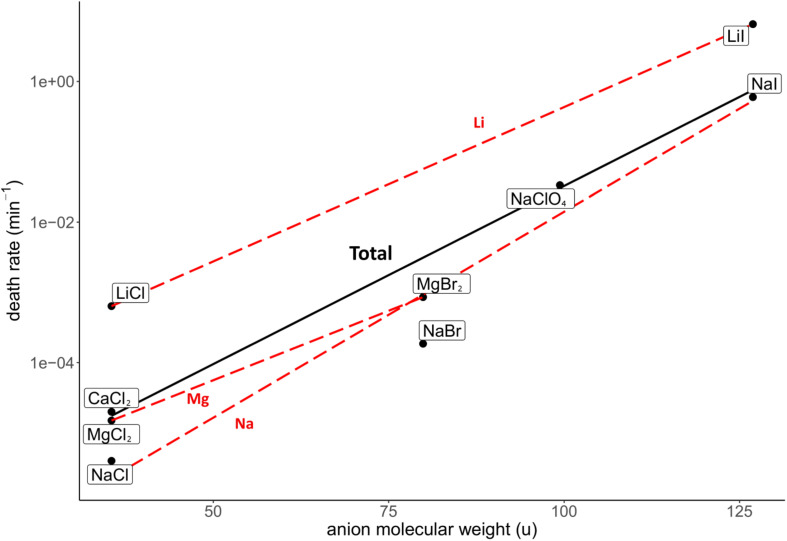
Correlation between the death rate at eutectic concentration and molecular weight of the anion of the respective salts. Regression of all salts is depicted in black (*R*^2^ = 0.85). Regressions of salts with the same cations are depicted with red dashed lines. The overall strong, positive correlation is supported by the trends of salts with the same anion, which all show a positive trend.

The investigated salt concentration independent parameters were the HBL-ion, ion molecular weight, hydrated ion radius and unhydrated ion radius. Strong, positive correlations were observed between the HBL-anion and death rate (*R*^2^ = 0.62; Figure 5), and between the anion molecular weight and death rate (*R*^2^ = 0.85; Figure 6). Although the correlations had a low quality; a weak, negative correlation was observed between the death rate and the cation molecular weight, the unhydrated cation radius and the HBL-cation, which were supported by negative trends in salts with the same anions (*R*^2^ = 0.26, 0.04 and 0.06, respectively) (Supplementary Figures S1–S3). A strong, positive correlation was observed between the death rate and the unhydrated anion radius (*R*^2^ = 0.34; Supplementary Figure S4). This is the opposite of the trends observed in the unhydrated cation radius. The negative correlation between the HBL-cation and death rate (Supplementary Figure S3) is contradictory to the HBL-anion results as described earlier (Figure 5). No correlation was observed between the death rate and the hydrated cation and anion radius (*R*^2^ = 0.10 and 0.01, respectively; Supplementary Figures S5, S6).

### Experiments at Non-eutectic Concentrations

**FIGURE 7:**
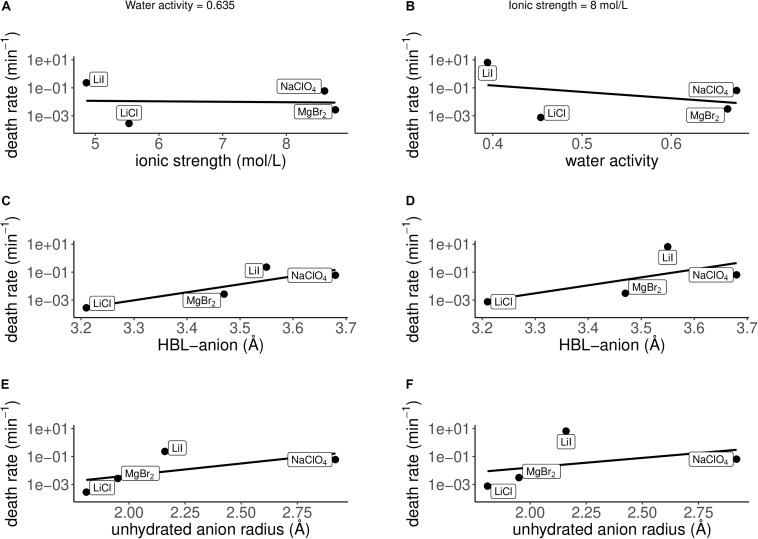
Correlations between the death rate and several physicochemical parameters at non-eutectic conditions: constant water activity at −15°C = 0.635 ± 0.02 **(A,C,E)**, constant ionic strength = 8 mol/L **(B,D,F)**. **(A)** No correlation is observed between the death rate and the ionic strength at a water activity at −15°C of 0.635 (*R*^2^ = 0.003). **(B)** A strong, negative correlation is observed between the death rate and the water activity at −15°C at an ionic strength of 8 mol/L (*R*^2^ = 0.14). **(C)** A strong, positive correlation is observed between the death rate and the hydration bond length between the anion and the oxygen atoms of the water molecules in the inner hydration shell (HBL-anion) at a water activity at −15°C of 0.635 (*R*^2^ = 0.75). **(D)** A strong, positive correlation is observed between the death rate and the hydration bond length between the anion and the oxygen atoms of the water molecules in the inner hydration shell (HBL-anion) at an ionic strength of 8 mol/L (*R*^2^ = 0.42). **(E)** A weak, positive correlation is observed between the death rate and the unhydrated anion radius at a water activity at −15°C of 0.635 (*R*^2^ = 0.41). **(F)** A weak, positive correlation is observed between the death rate and the unhydrated anion radius at an ionic strength of 8 mol/L (*R*^2^ = 0.15).

Apart from the eutectic concentrations, the death rate of *P. halocryophilus* has also been investigated for several salts (LiCl, LiI, MgBr_2_ and NaClO_4_) at salt concentrations with either a constant water activity of 0.635 ± 0.02 at −15°C (Figures 7A,C,E), or a constant ionic strength of 8 mol/L (Figures 7B,D,F). Death rate and physicochemical salt parameter values at at non-eutectic concentration at −15°C are presented in Table 2. The quality of the logarithmic regression of CFU counts to determine the death rate at non-eutectic concentrations was high, as *R*^2^ was higher than 0.8 for all salts (data not shown). At a water activity (at −15°C) of 0.635 ± 0.02, no correlation between the death rate and the ionic strength was found (*R*^2^ = 0.003; Figure 7A), which is consistent with the results at eutectic concentrations. At an ionic strength of 8 mol/L, a strong, negative correlation with low quality (*R*^2^ = 0.14) with the water activity at −15°C was observed (Figure 7B), which is consistent with the results at eutectic concentrations. Both conditions (constant water activity and constant ionic strength) showed similar results with each of the salt concentration independent salt parameters. The HBL-anion showed a strong, positive correlation at constant water activity (*R*^2^ = 0.75; Figure 7C) and at constant ionic strength (*R*^2^ = 0.42; Figure 7D), which is consistent with the results at eutectic concentrations. The unhydrated anion radius had a weak, positive correlation at a constant water activity at −15°C (*R*^2^ = 0.41) and with a lower quality at constant ionic strength (*R*^2^ = 0.15), respectively (Figures 7E,F), which is consistent with the results at eutectic concentrations. All other correlations with salt parameters at a water activity of 0.635 at −15°C (Supplementary Figure S7) and at an ionic strength of 8 mol/L (Supplementary Figure S8) had a low quality in both conditions (*R*^2^ ≤ 0.15).

## Discussion

### Physicochemical Effects

We found correlations of higher quality (*R*^2^ > 0.4) between several physicochemical parameters with the death rate of *P. halocryophilus*. These parameters are the salt concentration, HBL-anion and molecular weight of the anion. Correlations with a lower quality (*R*^2^ between 0.2 and 0.4) were found between the death rate and the water activity at −15°C, unhydrated anion radius, and molecular weight of the cation, some of which only became apparent by examining salts with the same cation or anion. The correlations of the following parameters were considered unreliable as they had a low quality (*R*^2^ ≤ 0.15) and are therefore interpreted as no correlations; the ionic strength, hydrated and unhydrated cation radius, hydrated anion radius and HBL-cation. The correlations at eutectic concentrations were confirmed by the correlations at non-eutectic concentrations. The parameters systematically affecting the death rate are related to each other according to the HSAB theory.

Based on the HSAB theory (Pearson, 1968a), salts consisting of hard cations and hard anions or soft cations and soft anions are less soluble than salts consisting of hard cations and soft anions or vice versa. In this study all salts contain hard cations and single-charged anions ranging from hard to soft (Pearson, 1968b). Chlorine is considered hard, bromine borderline and iodine soft (Pearson, 1968b). The increase in anion softness correlates with an increase of the unhydrated anion radius and the anions’ molecular mass. Increasing the anion softness in combination with the hard cations enhances the salts’ solubility, resulting in higher eutectic concentrations. Although soft ions have a low charge density, and therefore less water molecules in the hydration sphere, the combination of a soft anion with a hard cation results in a higher solubility, which in turn decreases the water activity, as more water molecules are needed to dissolve the increased number of ions in the eutectic solution. This is the case for the tested salts at eutectic concentrations for the salt concentration and the measured water activity, except for NaBr and NaI, which have similar water activity values (Table 1). The relation between those parameters explains the observation of the positive correlation between the death rate and the unhydrated anion radius (Supplementary Figure S6), molecular anion weight (Figure 6) and salt concentration (Figure 4), while the water activity at −15°C shows a negative correlation with the death rate (Figure 2).

A correlation between the survival, i.e., the inverse of the death rate, and water activity is expected, as a low water activity correlates with a decreased percentage of free biologically available water molecules (Tosca et al., 2008; Stevenson et al., 2015a, b). All measured water activity values are lower than calculated water activity values of these solutions at +25°C (Tables 1, 2). At lower temperatures, hydration shells are larger than at higher temperatures (Zavitsas, 2005; Heinz et al., 2018), resulting in a lower water activity of the surrounding solution. Although a lower water activity correlates to a lower survivability, microbial survival increases by lowering the temperature of brines (Heinz et al., 2018). This insight indicates that the correlation between water activity, temperature and survival is more complex than previously thought and further research is recommended.

Our results indicate that the ionic strength does not influence survival (Figure 3). In contrast, Fox-Powell et al. (2016) showed ionic strength to limit bacterial growth. A potential explanation for the observed differences between these studies could be that our experiments investigated a smaller range of ionic strength (4.9–11.7 mol/L) than Fox-Powell et al. (2016) (0–14 mol/L). Ionic strengths higher than 11.7 mol/L could have a negative effect on the survival of *P. halocryophilus* in the brines tested in the current paper. The lowest ionic strength that did not support growth in Fox-Powell et al. (2016) was 10.1 mol/L, while a different brine with an ionic strength of 12.1 mol/L did support microbial growth. Our research has only investigated one salt solution with an ionic strength larger than 10.1 mol/L, CaCl_2_ at a eutectic concentration. Hence, further research on the effect of high ionic strengths on the survival of *P. halocryophilus* is needed.

Harder ions form shorter and stronger hydrogen bonds due to higher charge densities, resulting in more water molecules surrounding the ion. Hence, hydration shells around hard ions are stronger and larger, as more water molecules surround the ion and are more strongly bound to the ion. The effect of the hydration shell strength on the survival of *P. halocryophilus* had previously been proposed by Heinz et al. (2018). A potential cause for this putative correlation is that the ion’s hydration shell is removed partially or completely by ion transport proteins prior to membrane transportation (Zhou et al., 2001; Gouaux and Mackinnon, 2005). Hence, stronger hydration shells (and thus shorter HBLs) require more energy for their removal from the ions and would therefore not be transported into the cell as much and as effectively as ions with longer HBLs. Stronger hydration shells would therefore correlate with a higher survival, i.e., lower death rate, which corroborates our observations of the hydration bond length of the anions (HBL-anion). However, our observations of the HBL-cation are not consistent with this hypothesis. There is a weak, negative correlation of low quality present between the HBL-cation and death rate, which was supported by negative trends when comparing salts with the same anion (a shorter HBL-cation correlates to a higher death rate). A possible explanation for the HBL-cation observation could be the fact that cations are significantly more hydrated at −15°C than at ambient temperatures (Zavitsas, 2005, 2016) while anions are usually less hydrated than their cationic counterparts (Ji, 1997, pp. 112–139). Thus, cations might have more difficulty to enter bacterial cells at −15°C because of a reduced ion mobility and permeability, which correlates with a decrease in the overall ion-specific toxicity (Seifriz, 1949). This effect could be sufficiently strong resulting in the absence of a strong correlation of survival with HBL-cation, while a strong correlation was present with the HBL-anion. Similar to our finding of more relevant anion-associated factors than those of the cation, a recent study shows that anions have the most important role in determining the maximum salt concentration suitable for growth of *P. halocryophilus*, thus influencing its survival (Heinz et al., 2019).

### Non-physicochemical Effects

Additionally, non-physiochemical effects such as chemical reactivity or biochemical effects of the species-ion interactions could play a role in the survival of *P. halocryophilus*. It is generally observed that salt shock results in plasmolysis, which inhibits nutrient uptake and DNA replication, and triggers an ATP level increase in cells, leading to inhibition of macromolecular biosynthesis (Csonka, 1989). The presence of both monovalent and divalent ions are important for the stability of RNA structures, as monovalent ions encourage secondary structure formation of RNA, while divalent ions stabilize RNA structures by shielding electronegatively charged groups (Ramesh and Winkler, 2010). Moreover, a combination of mono- and divalent ions ensure stable tertiary structures (Ramesh and Winkler, 2010). Magnesium, for example, effectively stabilizes tRNA tertiary structures (Draper et al., 2005). Therefore, changes in the ratio of mono- and divalent ions could influence the stability of biomolecules like RNA. Another biochemical effect could be influenced by different ion transportation mechanisms into and out of the cell. Ion transport of for example Na^+^, Ca^2+^ and Cl^–^ over the cell membrane can occur actively and passively via membrane transporters (Maloney, 2002; Padan, 2009). Therefore, ion-specific transport regulation mechanisms could influence the effect different ions have on the survival of microorganisms.

Depending on the ion, the overall survival of an organism can be affected. Calcium ions have, for example, been shown to interact with EPS of sulfur-reducing bacteria (Braissant et al., 2007). Additionally, CaCl_2_ is known to deflocculate sludge by the removal of EPS (Kavitha et al., 2015). In this study, the removal of EPS could have left the bacterial cells more exposed to the salt by reducing clumping, as observed previously (Heinz et al., 2019). Our study shows that the death rate in CaCl_2_ is slightly higher than that in MgCl_2_ or NaCl at their eutectic concentrations, but more calcium-containing salts would need to be tested to check if this effect occurs in more salts.

Chloride and bromide have little toxic effects on microorganisms (Flury and Papritz, 1993; Serrano, 1996). Bromide has previously shown to be comparably toxic as iodide to single-cell organisms (Flury and Papritz, 1993). However, iodide is a mildly reducing agent and can therefore induce reduction reactions with cell components causing cell damage. Additionally, iodide solutions contain minor amounts of iodine (I_2_) capable of oxidizing cell components. Both types of redox reactions can be harmful to the cells and, hence, reduce their survival in iodide solutions. Furthermore, iodide has protein denaturing properties (Dzubiella, 2008). Indeed, our experiments showed an increased toxicity when comparing iodide-containing salts to non-iodide-containing salts. In eutectic concentrations for example, the death-rate in LiI is 6.6 min^–1^, while the death-rate in LiCl is only 6.4 × 10^–4^ min^–1^, while the molar eutectic concentrations are similar (Table 1).

Not much is known about the microbial toxicity of dissolved perchlorate ions. In fact, dissimilatory perchlorate reduction is a common metabolic pathway in bacteria (Bardiya and Bae, 2011) and archaea (Liebensteiner et al., 2013), and perchlorate can be tolerated in high concentrations by eukaryotes, e.g., the halotolerant yeast *Debaryomyces hansenii* can tolerate 2.4 M NaClO_4_ (Heinz et al., 2020). Furthermore, dissolved perchlorate ions are relatively inert and non-oxidizing due to kinetic barriers (Urbansky, 1998). Although sodium perchlorate was more damaging to the survival of *P. halocryophilus* than sodium chloride at eutectic concentrations, specific toxic parameters belonging to the perchlorate ion only might play a minor role and probably can be neglected.

Further research could decipher the effect of additional non-physicochemical parameters to bacterial survival and therefore elaborate the results of the currently investigated effects of physicochemical parameters.

### Implications

Even though the addition of salt to aqueous environments would result in an increased temperature range of liquid water, and therefore would result in more potential habitable environments on Earth as well as on other planets, high salt concentrations have shown to have a detrimental effect on – even halotolerant – microorganisms. Our data shows that the survival of *P. halocryophilus* is affected by salinity and correlates simultaneously to multiple parameters which are chemically linked to each other. The water activity, salt concentration, hydration shell strength of the anion, anion molecular weight and unhydrated anion radius have shown to have the strongest correlation with the survival of *P. halocryophilus* in brines.

This study presents measured water activities for a large number of liquid salt solutions at constant sub-zero temperatures (−15°C) for the first time. As there are few datasets of water activity measurements in sub-zero liquid solutions (Toner and Catling, 2016), the dataset from this study gives a new, important insight of water activities in cryobrine environments relevant for planets such as Earth and Mars.

Together with water activity and salt concentration, the type of anion is the limiting factor for the survival of *P. halocryophilus* at low temperatures, and their toxicity is in turn correlated to their HBL, anion molecular weight and unhydrated radius. Thus, these parameters have the greatest influence on the habitability of saline environments, such as the abundant hygroscopic, saline environments and expected cryobrines on Mars, including the recently discovered putative subglacial lake (Orosei et al., 2018). These cryobrines haven been proposed to contain mainly chloride and perchlorate anions (Chevrier et al., 2009; McEwen et al., 2011). As *P. halocryophilus* has shown to have a high resistance to chloride and perchlorate containing brines (Heinz et al., 2019), this increases the probability of microbial life thriving in these environments. However, our results have shown that perchlorate containing salts are more damaging to the survival than chloride containing salts. This stresses the importance of anion brine composition, next to the importance of the salt concentration and water activity, when investigating the habitability of these brines. Hence, future Mars missions would need to take these environmental factors into account when investigating potential habitable environments.

## Data Availability Statement

The datasets generated for this study are available on request to the corresponding author.

## Author Contributions

AW and JH carried out the experimental work. AW performed the data analysis and wrote the first draft of the manuscript. All authors contributed to conception and design of the study, manuscript revision, and read and approved the submitted version.

## Conflict of Interest

The authors declare that the research was conducted in the absence of any commercial or financial relationships that could be construed as a potential conflict of interest. The reviewer AS declared a shared affiliation with no collaboration, with one of the authors AW, to the handling editor at the time of review.
