# Supplementary material for: Physicochemical Salt Solution Parameters Limit the Survival of Planococcus halocryophilus in Martian Cryobrines
Source: Front Microbiol. 2020 Jul 7;11:1284. doi: 10.3389/fmicb.2020.01284 (PMC7358355; doi:10.3389/fmicb.2020.01284)
Supplement: Supplementary file 1 [file Data_Sheet_1.docx]

Supplementary Material


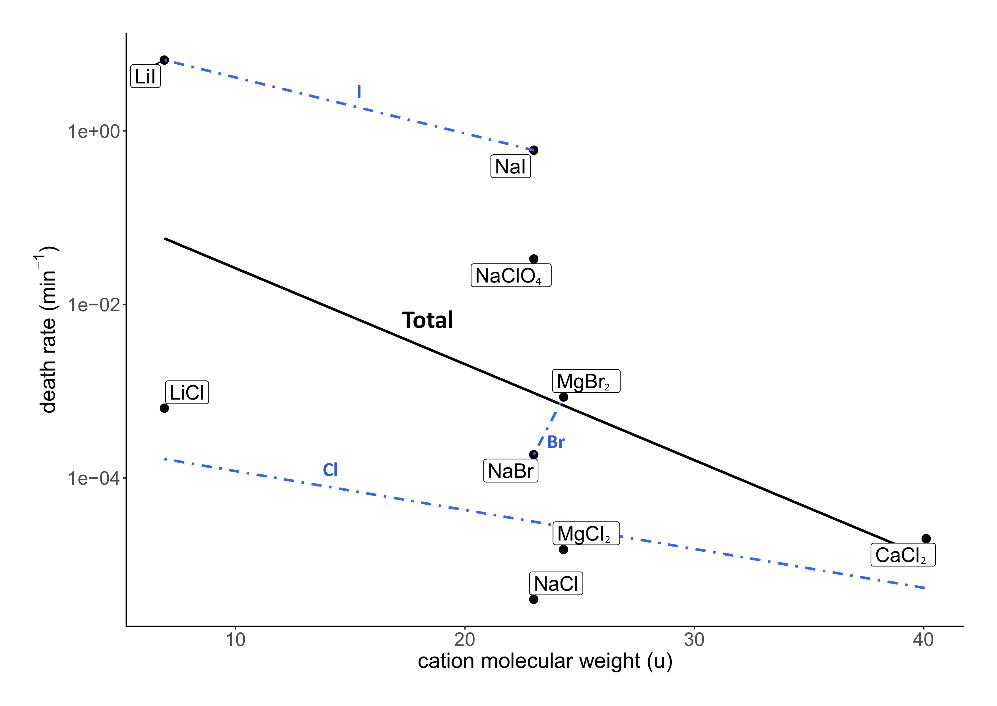


**Supplementary Figure 1.** Correlation between the death rate and molecular weight of the cation of the respective salts. Regression of all salts is depicted in black (R^2^ = 0.26). Regressions of salts with the same anions are depicted with blue dashdotted lines. A weak, negative correlation is seen, but only two out of the three trend lines of salts with the same anion show an indication of a negative correlation.


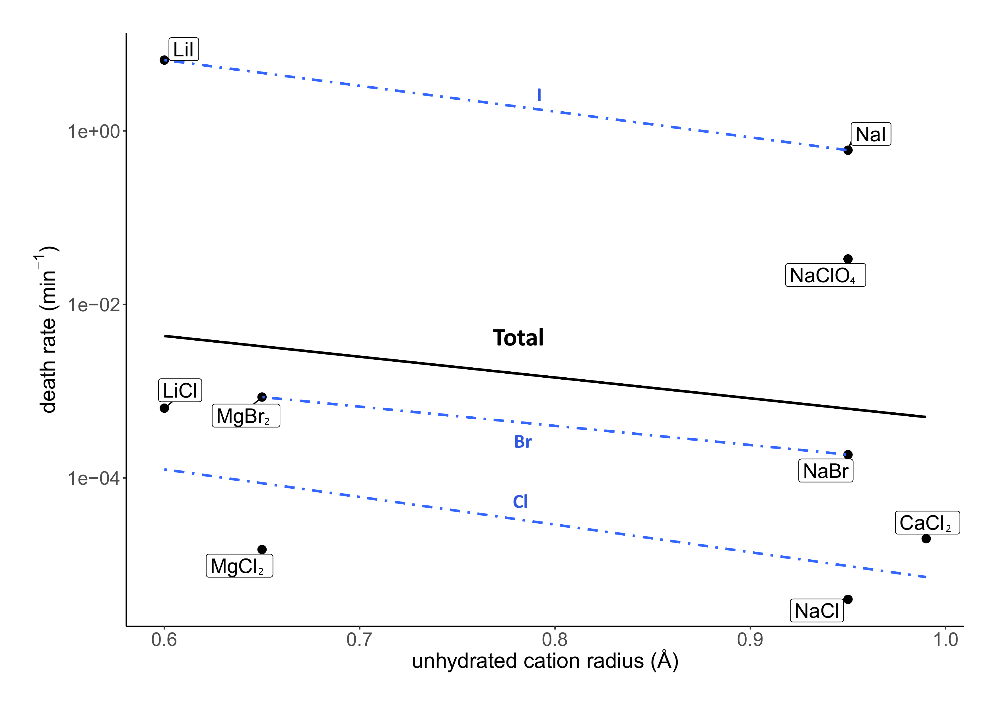


**Supplementary Figure 2.** Correlation between the death rate and unhydrated cation radius of the respective salts. Regression of all salts is depicted in black (R2 = 0.04). Regressions of salts with the same anions are depicted with blue dashdotted lines. A weak, negative overall correlation is seen, which is supported by the trend lines of salts with the same anion.


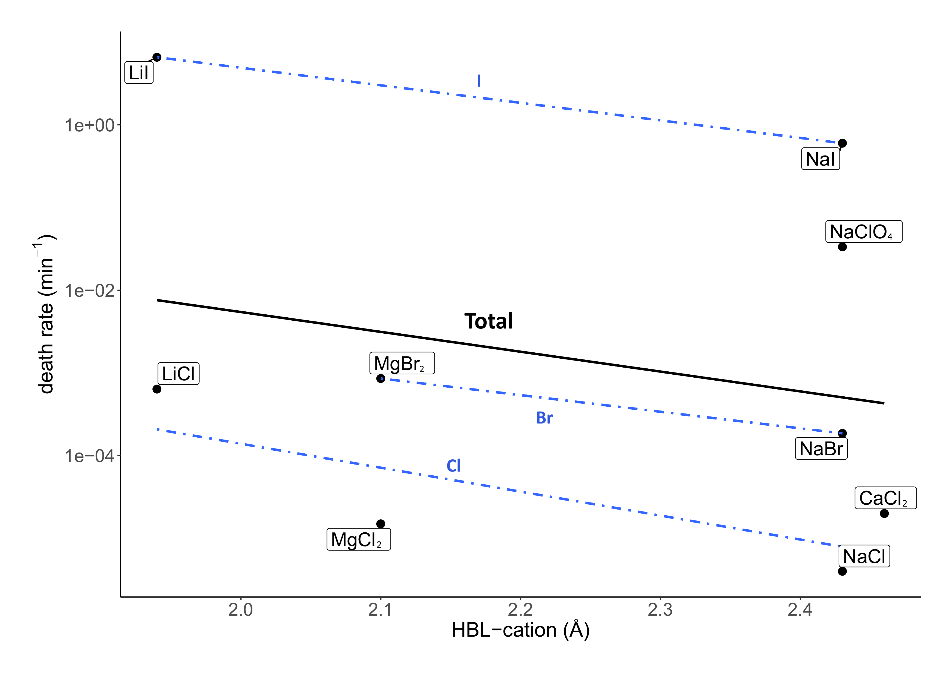


**Supplementary Figure 3.** Correlation between the death rate and hydration bond length between the cation and the oxygen atoms of the water molecules in the inner hydration shell (HBL-cation). Regression of all salts is depicted in black (R2 = 0.06). Regressions of salts with the same anions are depicted with blue dashdotted lines. A weak, negative overall correlation is seen, which is supported by negative trend lines of salts with the same anion.


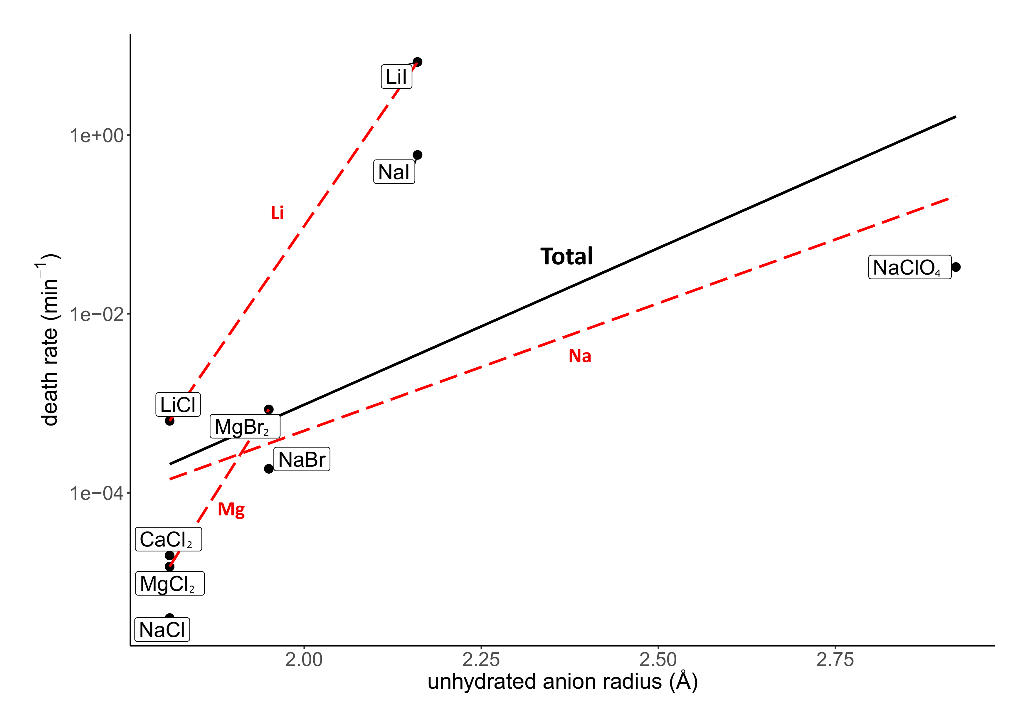


**Supplementary Figure 4.** Correlation between the death rate and unhydrated anion radius of the respective salts. Regression of all salts is depicted in black (R^2^ = 0.34). Regressions of salts with the same cations are depicted with red dashed lines. The overall strong, positive correlation is supported by the trends of salts with the same cation, which all show a positive trend.


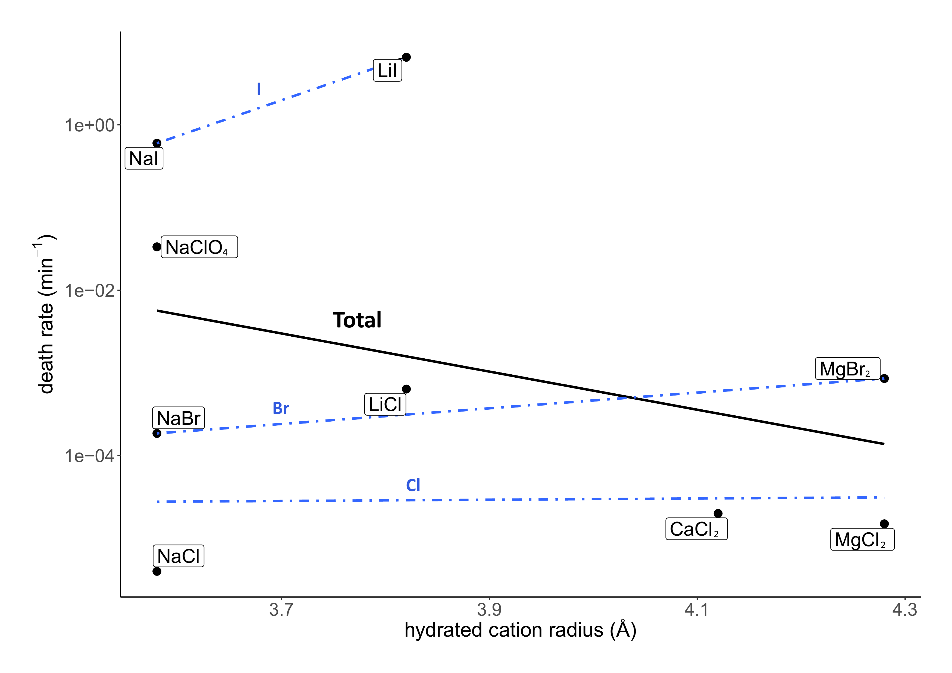


**Supplementary Figure 5.** Correlation between the death rate and hydrated cation radius of the respective salts. Regression of all salts is depicted in black (R^2^ = 0.10). Regressions of salts with the same anions are depicted with blue dashdotted lines. No overall correlation is seen, nor do the trend lines of salts with the same anion show an indication of a correlation.


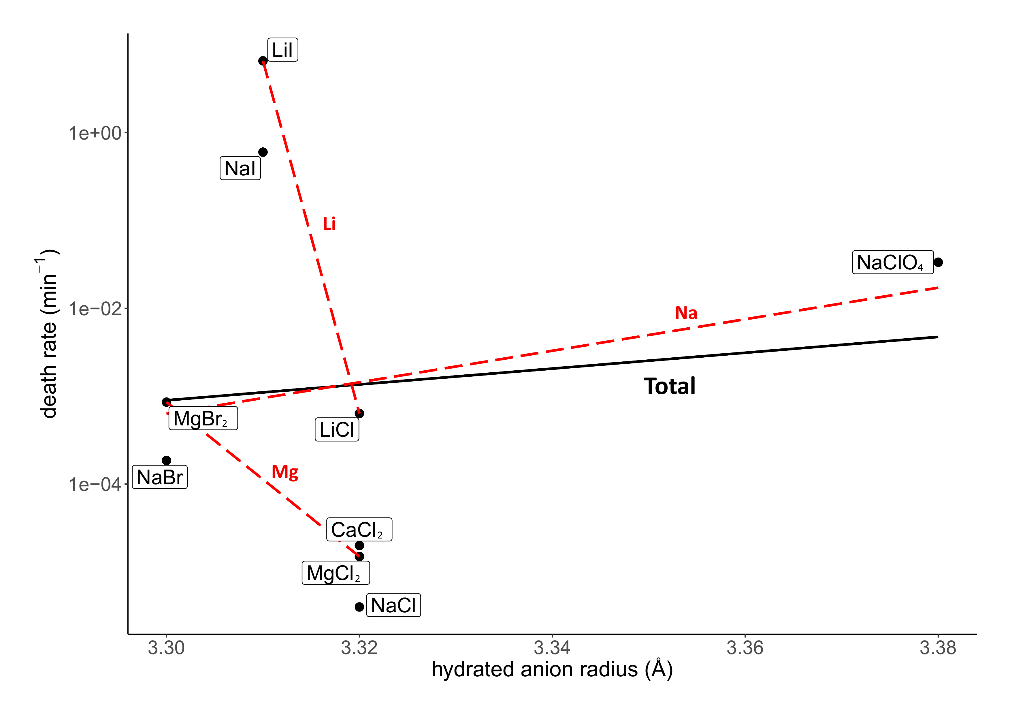


**Supplementary Figure 6.** Correlation between the death rate and hydrated anion radius of the respective salts. Regression of all salts is depicted in black (R^2^ = 0.01). Regressions of salts with the same cations are depicted with red dashed lines. No overall correlation is seen, nor do the trend lines of salts with the same cation show an indication of a correlation.


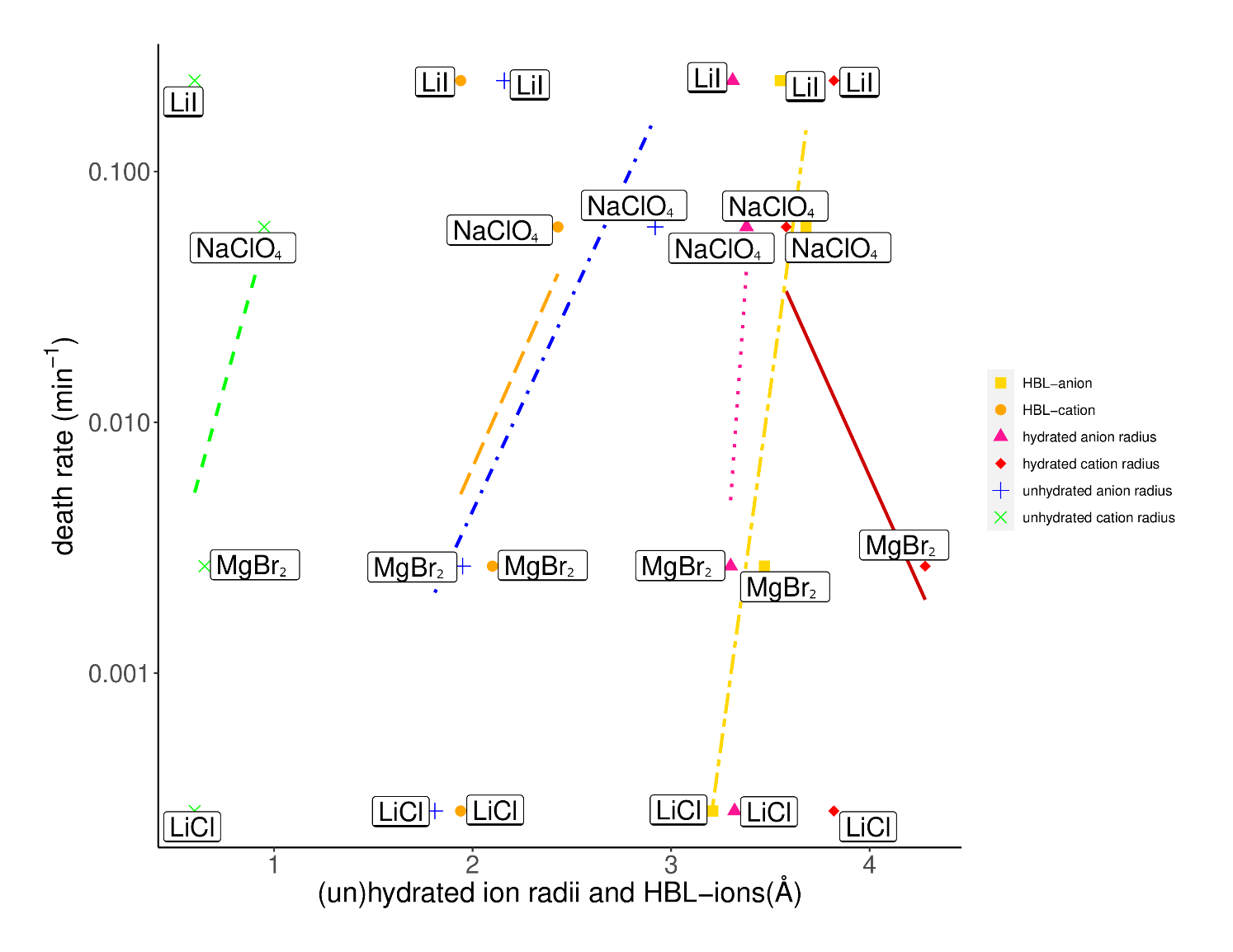


**Supplementary Figure 7.** Overview of correlations between the death rate and salt-concentration independent parameters at constant water activity = 0.635 + 0.02. A positive correlation is found between the death rate and the HBL-anion (R^2^ = 0.75), see yellow twodashed regression line. A positive correlation is found between the death rate and the HBL-cation (R^2^ = 0.10), see orange longdashed regression line. A positive correlation is found between the death rate and the hydrated anion radius (R^2^ = 0.10), see pink dotted regression line. A negative correlation is found between the death rate and the hydrated cation radius (R^2^ = 0.15), see red solid regression line. A positive correlation is found between the death rate and the unhydrated anion radius (R^2^ = 0.41), see blue dashdotted regression line. A positive correlation is found between the death rate and the unhydrated cation radius (R^2^ = 0.13), see green loosely dashed line.


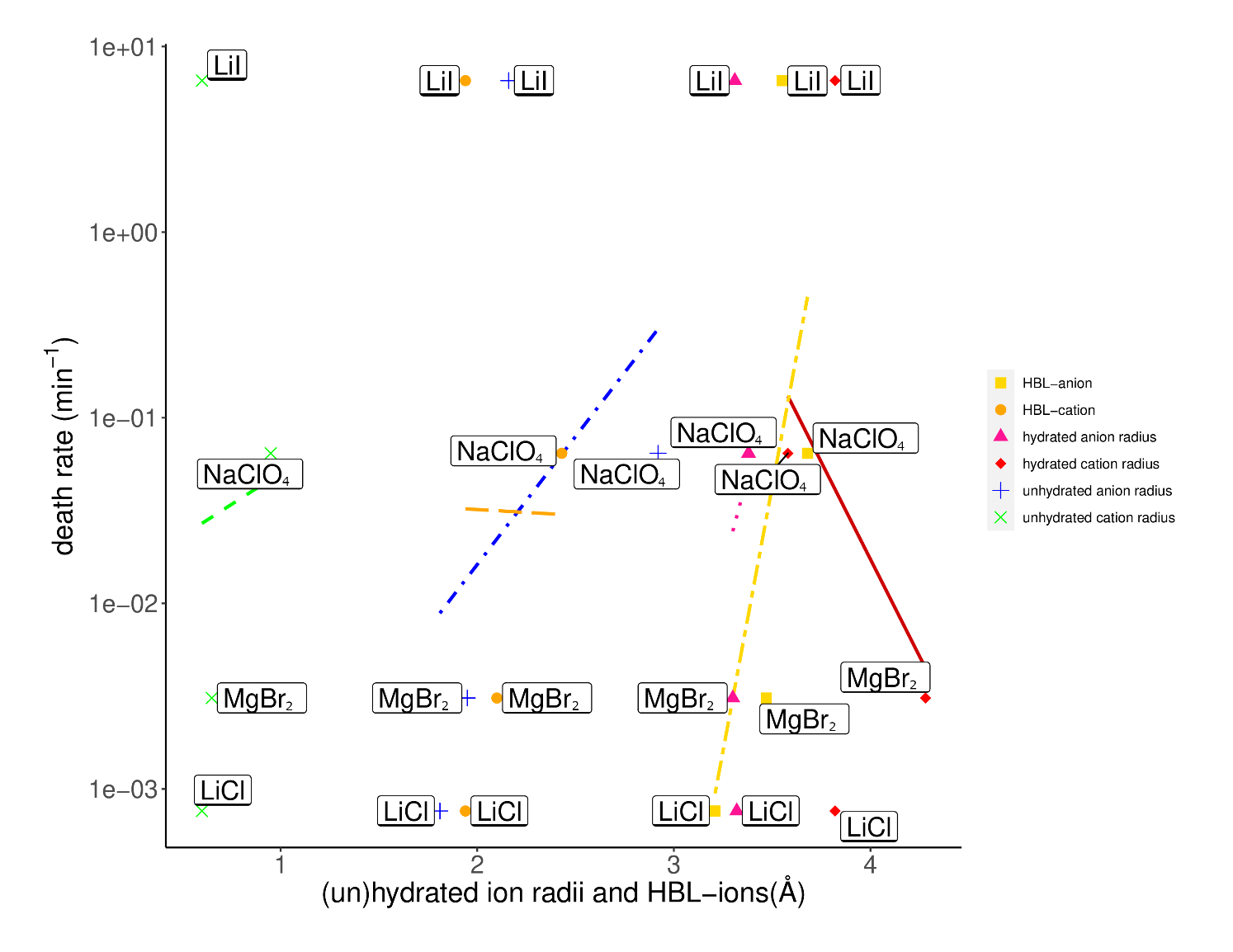


**Supplementary Figure 8.** Overview of correlations between the death rate and salt-concentration independent parameters at constant ionic strength = 8 mol/L. A positive correlation is found between the death rate and the HBL-anion (R^2^ = 0.42), see yellow twodashed regression line. No correlation is found between the death rate and the HBL-cation (R^2^ = 0.00), see orange longdashed regression line. A positive correlation is found between the death rate and the hydrated anion radius (R^2^ = 0.01), see pink dotted regression line. A negative correlation is found between the death rate and the hydrated cation radius (R^2^ = 0.12), see red solid regression line. A positive correlation is found between the death rate and the unhydrated anion radius (R^2^ = 0.15), see blue dashdotted regression line. A positive correlation is found between the death rate and the unhydrated cation radius (R^2^ = 0.04), see green loosely dashed line.
